# Extending section 12 approval under the Mental Health Act to professions other than medicine

**DOI:** 10.1192/bjb.2024.7

**Published:** 2024-12

**Authors:** John L. Taylor, Carole Burrell

**Affiliations:** 1 Northumbria University, Newcastle upon Tyne, UK; 2 Cumbria, Northumberland, Tyne and Wear NHS Foundation Trust, Newcastle upon Tyne, UK

**Keywords:** Section 12 approval, England and Wales Mental Health Act, deprivation of liberty safeguards, approved clinicians, objective medical expertise

## Abstract

Applications for detention under civil sections of the England and Wales Mental Health Act 1983 require at least one recommendation from a registered medical practitioner who is approved under section 12 of the Act. The Mental Health Act 2007 introduced multi-professional approved clinicians who may act as a patient's responsible clinician with responsibilities that include renewal of detention for treatment. Approved clinicians who are medical practitioners are automatically approved for section 12 purposes, whereas other approved clinicians are not. It is argued in this paper that this inconsistency is illogical, has implications for patient care and needs to be remedied.

The UK government published a draft Mental Health Bill concerning reforms to the England and Wales Mental Health Act 1983 (the ‘Act’) in July 2022.^1^ The draft Bill was preceded by the Independent Review of the Mental Health Act 1983, chaired by Professor Sir Simon Wessely, which made 154 recommendations for reforming the Act in its final report.^2^ One area of existing legislation that was not covered by the Independent Review or addressed in the draft Bill is the eligibility criteria for those practitioners providing recommendations under section 12 for applications for the detention of patients for assessment or treatment under Part II (civil) sections of the Act.

## Current legislative position

Other than in the case of an emergency admission, when the Act requires only one recommendation, the legal requirement is for two medical recommendations, one of which can be made only by a registered medical practitioner who is approved under section 12 as having ‘special experience in the diagnosis or treatment of mental disorder’.^3^ Somewhat surprisingly given the requirement for this ‘special experience’ in the primary legislation, the secondary legislation concerning the approval of section 12 doctors allows for general practitioners who may not have any specialist knowledge or training in mental health to be approved under this section.^4^

Under subsection 2A of section 12, ‘A registered medical practitioner who is an approved clinician shall be treated as also approved for the purposes of this section [ … ] as having special experience [in the diagnosis or treatment of mental disorder]’, so that all medical practitioners who are considered to have the requisite professional requirements to be approved as approved clinicians are automatically added to the section 12 approvals database.

## The Mental Health Act 2007 and objective medical expertise

Article 5 of the European Convention on Human Rights (ECHR)^5^ concerns the right to liberty and security and states that no one should be deprived of their liberty except in certain circumstances and following due legal process. Article 5(1)(e) allows for people to be lawfully detained on the basis of ‘unsound mind.’ The European Court of Human Rights (ECtHR) found in the case of *Winterwerp v Netherlands* [1979]^6^ that in order to be lawful, detention (and continued confinement) on grounds of unsoundness of mind requires ‘objective medical expertise’ to establish that the person has a true mental disorder.

The Mental Health Bill 2006 – which preceded the amending Mental Health Act 2007 – proposed the introduction of approved clinicians who need not be medically qualified but would be able to renew detention under the Mental Health Act. During its scrutiny of the Bill, the UK Parliamentary Joint Committee on Human Rights argued that to be ECHR compliant, the objective medical expertise requirement necessitates the opinion of a medically qualified expert.^7^ In making its case, the Joint Committee relied on *Varbanov v Bulgaria* (2000),^8^ in which the ECtHR held that it is a breach of Article 5 if a person is detained, or continues to be detained, owing to unsound mind without the opinion of a medical expert – which the Committee took to mean a psychiatrist. In fact, in the case of *Varbanov*, the ECtHR found that a prosecutor's or police examination did not provide the authority for compulsory detention and contrasted this with an assessment by a psychiatrist.

On this basis, the UK government did not agree with the Joint Committee that *Varbanov* (or *Winterwerp*) required a psychiatrist to provide the necessary medical expertise. It argued that there is no specific case law that defines what is meant by medical expertise and, within a modern workforce, it is appropriate for Mental Health Act functions to be carried out by those who are competent to perform them.^7^

The eminent lawyers Richard Gordon KC^9^ and Richard Jones^10^ agreed with the government's view on the basis that any professional detaining or renewing detention must demonstrate to a delegated authority (i.e. an Approvals Panel) their competency in identifying the presence and severity of a mental disorder. Thus, it is likely that the ECtHR would find the government's proposal to approve non-medically but highly qualified mental health professionals with the relevant competencies as approved clinicians with the power to renew detention to be ECHR compliant.

## Deprivation of liberty safeguards and objective medical expertise

More recently, in the context of reform of the deprivation of liberty safeguards under the Mental Capacity Act 2005, the Law Commission considered the issue of objective medical expertise in relation to deprivation of liberty on the basis of unsoundness of mind. The Commission's view was that ‘The Strasbourg court has not stated clearly what qualifications or competencies the state should require of medical experts’ (para. 7.173), even if some case decisions could be interpreted as suggesting that the medical expert must be a psychiatrist.^11^

In its final report to parliament, the Commission stated that ‘it would be highly unlikely that any court today would interpret Article 5 as laying down a general rule that objective medical expertise can only be provided by a psychiatrist, or even a doctor’ (para. 9.66)^12^ and existing ECtHR (e.g. *Ruiz Rivera v Switzerland*, 2014)^13^ and domestic case law (e.g. *G v E and others*, 2010)^14^ supports this view. That it is for national authorities to determine which professional qualifications are required in order to provide objective medical expertise is reaffirmed in the ECtHR judgment in the case of *Ilnseher v Germany* (2018).^15^ While acknowledging that in certain cases, such as where the assessment concerns a person with no prior history of mental disorder, the medical expert needs a ‘specific qualification’, the judgment stated that ‘in general [the] national authorities are better placed than itself to evaluate the qualifications of the medical expert in question’ (para. 130).^15^

This view is reflected in the draft regulations – set out in a Department of Health and Social Care consultation in 2022 – that support the Mental Capacity (Amending) Act 2019. The draft regulations concerning deprivation of liberty assessments, determinations and pre-authorisation reviews stipulate that ‘medical assessments’ to determine the presence of a mental disorder can be carried out by registered medical practitioners and registered psychologists.^16^

## The introduction of approved clinicians

The Mental Health Act 2007, which amended the 1983 Act, introduced the role of the approved clinician, an individual who is approved by a delegated authority of the Secretary of State. Psychologists, nurses, social workers and occupational therapists, as well as medical practitioners, are eligible under the 2007 Act to be approved clinicians and thus act as the responsible clinician with overall legal responsibility for patients subject to the provisions of the 1983 Act. Responsibilities reserved to the responsible clinician include renewing a patient's detention, placing a patient on a community treatment order (CTO) and discharging a patient from detention or a CTO.

The secondary legislation concerning the approval of persons to act as approved clinicians in England is set out by the Secretary of State in instructions published 2015.^17^ To be approved an applicant must provide evidence to demonstrate to the approving body's satisfaction that they have the relevant competencies, including under the Assessment competency the ‘Ability to – (a) identify the presence of mental disorder; (b) identify the severity of the mental disorder; and (c) determine whether the mental disorder is of a kind or degree warranting compulsory detention’ (p. 8).^17^ In terms of the Treatment competency, the applicant must demonstrate an ‘Understanding of – (a) mental health related treatments, which include physical, psychological and social interventions; (b) different evidence based treatment approaches and their applicability to different patients; and (c) the range of appropriate treatments and treatment settings which can be provided in the least restrictive environment and will deliver the necessary health and social outcomes’ (p. 8).^17^ In Wales, the Welsh Ministers set out the competencies that approved clinicians must be able to demonstrate in subordinate legislation in 2018.^18^

## Lack of parity and its consequences

Given these competency requirements, it can be argued that all approved clinicians possess the ‘special experience in the diagnosis or treatment of mental disorder’ required under section 12 – in the same way that under subsection 2A of section 12 a registered medical practitioner who is an approved clinician is automatically approved for the purposes of this section 12. On the face of it, it seems anomalous that approved clinicians who are not medical practitioners cannot be approved under section 12 to provide the recommendations required for a person to be detained for assessment or treatment under Part II provisions of the Act, although they can renew detention under these provisions, applying precisely the same criteria concerning the presence of a mental disorder requiring detention in hospital for the purposes of assessment or treatment that is necessary for health and safety of the person or for the protection of others.

As it stands, the law concerning professional eligibility for section 12 approval is out of kilter with a modern mental health service incorporating new ways of working and extended roles under the Act^19^ and can lead to perverse situations. For example, an approved clinician who is not a medical practitioner could be the responsible clinician for a patient detained for assessment under section 2 of the Act. Following a period of in-patient assessment it is agreed that the patient should undergo a further Mental Health Act assessment for detention for treatment under section 3. As things are, the patient's responsible clinician is unable to provide a recommendation for the application for section 3 detention and two medical practitioners, who may have minimal or no prior knowledge of the patient, would have to be drafted in to provide the required recommendations. This is a scenario experienced by the first author (J.L.T.) recently.

The ongoing exclusion of approved clinicians who are not medical practitioners from section 12 eligibility is all the more surprising given the workforce challenges in implementing the proposed reforms to the Act in general, and in relation to accessing timely Mental Health Act assessments in particular. Sourcing section 12 doctors is one significant reason for severe delays in these assessments being completed, which is putting patients and their families at increasing risk.^20^ This issue was addressed also by the Independent Review of the Act,^2^ which found that problems with getting assessments completed by section 12 doctors were leading to undue delays. It was reported that ‘many parts of the country struggle to find doctors who can and are willing to perform this function’, there is an over-reliance on retirees, and approved mental health professionals were often left to ‘dial around’ looking for section 12 doctors over the telephone (p. 217).^2^ The Review recommended that this issue should be reviewed and addressed. Although the number of approved clinicians who are not medical practitioners is low currently, the NHS long-term workforce plan^21^ includes the training of more than 1000 additional approved clinicians over the next 8 years, with a focus on multi-professional approved clinicians to ‘improve access to services and quality of care’ (para. 4.97). If these additional approved clinicians were also approved for the purposes of section 12 then they could assist with the current difficulties in accessing section 12 doctors for Mental Health Act assessments.

## Conclusions

It is time for the legislation to be amended so that all approved clinicians are treated as also approved for the purposes of section 12 as having special experience in the diagnosis or treatment of mental disorder. Patients – particularly those with complex biopsychosocial problems – would benefit from multi-professional approved clinicians being able to make recommendations about initial detention. Further, such a change could help with the provision of more timely access to section 12 approved clinicians to carry out Mental Health Act assessments and thus assist patients who often spend too long, frequently in distressed and disturbed states, in inappropriate settings such as police cells, awaiting these assessments.

One issue that could arise in approving approved clinicians who are not medical practitioners under section 12 is the exclusion of physical diseases that might be underlying a patient's presentation during Mental Health Act assessments. Currently, section 12 approval does not require expertise in physical disease, and any application for detention under the Act relies on the identification of a mental disorder that requires such detention in the interests of the patient's safety or for the protection of others, irrespective of the underlying causes of that disorder. Given that statutory guidance^22^ is that one recommendation for detention should always be provided by an assessor with previous knowledge of the patient, and in practice at least one of the section 12 assessors will invariably be a medical practitioner (and the code of practice could make this a requirement), this issue should not be a significant obstacle.

The government is yet to respond to the report of the joint parliamentary scrutiny committee on the draft Mental Health Bill^23^ or set a timetable for the implementation of a new Mental Health Act, so there is time for changes concerning section 12 approval to be considered and introduced as part of the overhaul and modernisation of this legislation – and harmonisation with the reformed Mental Capacity Act in relation to the assessment of mental disorder associated with deprivation of liberty.

## About the authors

**John L. Taylor** is a Professor in the Northumbria Law School at Northumbria University; a consultant clinical psychologist and approved clinician with Cumbria, Northumberland, Tyne and Wear NHS Foundation Trust, Newcastle upon Tyne, UK; and Chair of the Mental Health Act Advisory Group of the British Psychological Society. **Carole Burrell** is an Associate Professor in the Northumbria Law School at Northumbria University, Newcastle upon Tyne, UK.

## Data Availability

Data availability is not applicable to this article as no new data were created or analysed in this study.
